# Differential Nitrogen Cycling in Semiarid Sub-Shrubs with Contrasting Leaf Habit

**DOI:** 10.1371/journal.pone.0093184

**Published:** 2014-03-27

**Authors:** Sara Palacio, Melchor Maestro, Gabriel Montserrat-Martí

**Affiliations:** 1 Instituto Pirenaico de Ecología (IPE-CSIC), Avda. Nuestra Señora de la Victoria, Jaca, Huesca, Spain; 2 Instituto Pirenaico de Ecología (IPE-CSIC), Avda. Montañana, Zaragoza, Spain; US Geological Survey, United States of America

## Abstract

Nitrogen (N) is, after water, the most limiting resource in semiarid ecosystems. However, knowledge on the N cycling ability of semiarid woody plants is still very rudimentary. This study analyzed the seasonal change in the N concentrations and pools of the leaves and woody organs of two species of semiarid sub-shrubs with contrasting leaf habit. The ability of both species to uptake, remobilize and recycle N, plus the main storage organ for N during summer drought were evaluated. We combined an observational approach in the field with experimental ^15^N labelling of adult individuals grown in sand culture. Seasonal patterns of N concentrations were different between species and organs and foliar N concentrations of the summer deciduous *Lepidium subulatum* were almost double those of the evergreen *Linum suffruticosum*. *L. subulatum* up took ca. 60% more external N than the evergreen and it also had a higher N resorption efficiency and proficiency. Contrastingly, *L. suffruticosum* relied more on internal N remobilization for shoot growth. Differently to temperate species, the evergreen stored N preferentially in the main stem and old trunks, while the summer deciduous stored it in the foliage and young stems. The higher ability of *L. subulatum* to uptake external N can be related to its ability to perform opportunistic growth and exploit the sporadic pulses of N typical of semiarid ecosystems. Such ability may also explain its high foliar N concentrations and its preferential storage of N in leaves and young stems. Finally, *L. suffruticosum* had a lower ability to recycle N during leaf senescence. These strategies contrast with those of evergreen and deciduous species from temperate and boreal areas, highlighting the need of further studies on semiarid and arid plants.

## Introduction

After water, nitrogen (N) is the most limiting resource for plant growth in arid and semiarid ecosystems [1], [2]. N sources for plants can be both external and internal. The former include mineralization of the soil organic matter, microbial fixation of atmospheric N_2_, organic N transfers from mycorrhizal symbionts to plant roots and, in some areas, also atmospheric N deposition [3]. Internal N sources come from storage through remobilization and recycling [4]. They determine the proportion of N lost from the plant and also the ability of plants to survive in areas where N availability through external sources is low. Internal nutrient cycling could be particularly relevant in arid and semiarid areas, where the low, variable and unpredictable precipitation results in less productivity, subsequently smaller organic matter inputs to the soil and slower mineralization rates [5], [6], [7]. Furthermore, in semiarid areas where soils are rich in gypsum, calcium saturates the soil ion exchange complex, making the soil less fertile [8], [9]. Understanding how plants from semiarid and arid areas adjust their internal N cycling is hence crucial.

The internal N cycling of woody species is regulated by environmental cues and plant phenology. In temperate plants, allocation of N to storage is normally triggered by short days and cold temperatures [10], [11]. Accordingly, in temperate trees and shrubs, N stores are built up in autumn from both N recycled from senescing organs and external N uptake which can occur independently of canopy senescence [12], [13]. In most plants growing in Mediterranean and semiarid ecosystems, leaf senescence takes place at the onset of summer [14], [15], [16], so environmental cues regulating storage may be different. Stored N is subsequently remobilized in spring to supply new growth in a process that is source driven i.e. N stores are remobilized independently of the sink strength of the growing organs [4]. At this time of the year, new growing shoots tend to show maximal N mass-based concentrations that are subsequently diluted as the shoots develop [16], [17], [18]. Spring remobilization of stored N can take place before external N uptake starts [3]. It represents a highly variable proportion of the total N invested in woody plant growth that increases with N limitation [19], [20], [21], [22], indicating the relevance of N storage and remobilization for plant growth when soil N availability is low. Although we currently have a reasonably good understanding of the internal N cycling of trees and shrubs from temperate, boreal and Mediterranean systems [3], [14], [18], [23], [24], [25], [26], [27], information related to woody species from arid and semiarid ecosystems is particularly scarce, but see [16], [28], [29]. In particular, no previous studies have attempted to quantify the relative contribution of stored N to the N requirements for growth of woody species from arid or semiarid areas (see review by [3]).

Perennial plants growing in N-poor soils are adapted to maximizing external N-uptake and/or minimizing N losses through plant litter [30]. However, leaf longevity seems to affect the strategies displayed by different species, with evergreens being more efficient in reducing N losses either through efficient internal N recycling or by decreased biomass turnover (slow growth rates, low oscillations of living biomass, etc), while deciduous species seem to be more efficient in N uptake [28]. Plant leaf habit also determines the preferred site of N storage within the plant: whilst evergreen species (including conifers) store N preferentially in the foliage, deciduous plants store it in woody organs such as the root or trunk [3], [19], [27]. Further, the growth-form of species can affect the site of N storage and the N status of plants [31], [32]. Consequently, the effect of leaf longevity on the N cycling of plants should be assessed within species of the same growth-form [16], [19].

Woody species growing in semiarid environments are typically sub-shrubs with a high diversity of leaf longevity patterns [33]. Most of these plants are normally considered seasonally dimorphic species, i.e. plants that bear two different types of shoots throughout the year (short and long shoots) with different morphological and physiological characteristics [33], [34]. Nevertheless, leaf longevity can be extremely variable across seasonally dimorphic plants, ranging from species that lose most of their leaves during summer (i.e. summer deciduous species) to species shedding only a few leaves [15]. Such differences can have a strong influence on the N cycling of plants [16]. However, very few studies have addressed the implications of leaf longevity on the internal N cycling of plants from N-deprived semiarid environments. In particular, the differential allocation of N to storage in species with contrasting leaf habit remains unexplored.

In this study we analyze the internal N cycling in two co-existing seasonally dimorphic semiarid sub-shrubs with different leaf longevity: *L. subulatum* is a summer deciduous while *L. suffruticosum* retains part of its foliage during summer. We hypothesize that: 1) seasonal patterns of N concentrations and pools will differ between species owing to their different leaf habit but maximum foliar N concentrations will be reached in spring in the young leaves of both species, matching the period of long shoot elongation; 2) the summer deciduous species, *L. subulatum*, will show a higher ability to uptake N from external sources than the evergreen, while the latter will show a more proficient N recycling; and 3) *L. subulatum* will store N preferentially in the wood during summer while *L. suffruticosum* will do so in the old foliage.

## Materials and Methods

### Study species

The two species of sub-shrubs selected for study: *Linum suffruticosum* L. and *Lepidium subulatum* L., have similar height, leaf morphology and are seasonally dimorphic species but they differ in the degree of reduction of their transpiring biomass throughout the year and their specificity to gypsum soils [15], [35], [36]. While *L. suffruticosum* maintains some of its leaves during summer, *L. subulatum* dries out most of its leaves, hence reducing the amount of productive and transpiring biomass during summer drought [15]. *L. suffruticosum* is not exclusive to gypsum soils whilst *L. subulatum* is a widespread Iberian gypsum specialist [37]. None of the species included in this study are endangered or protected.

As all seasonally dimorphic plants, *L. suffruticosum* and *L. subulatum* display two different types of branches: short and long branches, and shoot growth occurs in two main pulses [36]. In both species the main growth pulse begins by mid February and lasts until the end of June, involving the growth of both long and short branches. The second one occurs during autumn, from September to November, and involves the development of short branches and a few long branches. In *L. subulatum*, flowering and fruiting begin in April and last until May and June respectively. *L. suffruticosum* delays fruiting and flowering one month, starting in May and concluding in June and July respectively.

### Design of the study

We combined an observational approach in the field with experimental ^15^N labelling of adult individuals grown in sand culture in pots. The first approach aimed at gaining information on the seasonal dynamics of N mass-based concentrations and pools in the different organs of both species growing in natural conditions, whilst experimental ^15^N labelling aimed at quantifying the relative contribution of remobilization from storage vs. external uptake to the growth of both species throughout a growing season. This latter approach also enabled identifying the main organ of N storage of both species and their ability to withdraw N from senescing organs.

### Seasonal dynamics of N concentrations and pools

Field records were taken in a nearly-pure gypsum hill in the gypsum outcrops of Villamayor, near Zaragoza, in NE Spain (41°42′44″N 0°43′59″W, at 320 m a.s.l; Table 1). Permit for sampling of plant material was issued by the Government of Aragón (Diputación General de Aragón, DGA) to GMM and SP. The dominant substratum in this area is gypsum, with a few thin inserted outcrops of marls and clays [38]. Almost-pure gypsum soils, such as the ones included in this study, are particularly stressful areas due to their low water retention and their low fertility [8]. Climate is semiarid and highly seasonal [35], with a mean annual temperature of 14.6°C, and an average annual rainfall of 334.5 mm, which falls mainly during spring and autumn [39]. At this site, these two species coexist and display a similar degree of abundance. For further information about this site see [36] and [35].

**Table 1:** Chemical composition: percentage of organic matter (OM), Nitrogen (N), Carbon (C), Gypsum and carbonates (CaCO_3_) of the soils included in the study.

Soil source	pH	% OM	% N	% Gypsum	%CaCO_3_
**Villamayor 1** †	7.7	1.9	0.14	55.5	15.6
**Villamayor 2** *	8.3	0.7	0.04	74.2	9.0
**Pots**	8.0	0.3	0.01	19.0	2.0

† Soil of the study site for the seasonal change in N concentrations in Villamayor, Huesca (41°42′44″N 0°43′59″W).

* Soil collected in Villamayor, Huesca (41°41′50″N, 0°44′42″W) and mixed in a 1∶5 proportion with inert silica sand to produce the substrate included in pots.

Sampling for total N analyses was conducted monthly between September 2002 and January 2004. On each occasion, five adult individuals of *L. suffruticosum* and *L. subulatum* were excavated up to 30–40 cm from their rooting point and only medium sized plants were selected. The excavated plants were separated into coarse roots (>5 mm in width), main and secondary stems (older than three-year-old), short shoots (whole), and current-year leaves and stems of the long shoots. In spring, a new cohort of long shoots was formed. From that time on, this new cohort was incorporated in the analyses. All woody plant material was vigorously brushed to remove soil particles, cut in small pieces, and stored at −20°C until freeze-dried (Cryodos, Telstar Industrial SL, Terrasa, Spain).

On each sampling date a three-year-old branch was sampled from each of 15 different individuals, randomly selected within the population for the measurement of branch biomass partitioning. In spring time, when the new shoots were expanding, four different cohorts of shoots coexisted in the three-year-old branches. In order to have comparable samples throughout the year, once spring growth had finished, our branch unit comprised only the three youngest cohorts of shoots. Branches were separated into their different components: stems and leaves of current-year long shoots, one-year and two-year-old long shoots (if available) and long shoots formed in the course of the study; current-year short shoots (including both stems and leaves) and short shoots formed in the course of the study; reproductive fractions and dry and senescent elements of the branch. Fractions were oven dried to a constant weight at 60°C and weighed to the closest 0.01 mg. To scale measurements up to the whole plant canopy, the average number of three-year-old branches per medium-sized individual was calculated by counting all the three-year-old branches in 15 medium-sized individuals of each species. The amount of N in the different fractions of the plants was calculated as the product between N mass-based concentrations (n = 5) and the average weight of each fraction per plant canopy (n = 15).

### Experimental ^15^N labelling

In late August 2009, when summer drought was at its highest and *L. subulatum* plants were leafless, five medium-sized adult individuals of each species were lifted from the same field site used for seasonal N measurements (see above). Plants with the soil attached to their roots were taken to the laboratory where they were carefully washed to remove all soil particles without the disruption of fine roots. Plants were then planted in a mixture of inert silica sand with gypsum soil at 5∶1 proportion (i.e. 20% gypsum soil) in 0.018 m^−3^ pots. The gypsum soil included in the mixture had been collected from the field in Villamayor, Zaragoza (41°41′50″N, 0°44′42″W), approximately 3 Km away from the field site used for seasonal N monitoring, and had a gypsum content of ca. 75% (see Table 1). The sand∶soil mixture was homogenized with a cement mixer. Pots were kept at ambient conditions in the grounds of the Pyrenean Institute of Ecology (CSIC) in Zaragoza (41°42′44″N 0°43′59″W), located ca. 8 km from the field site and hence with similar weather conditions. The pots received natural rainfall and were watered twice a week with 200 ml of a nutrient solution containing ^15^NO_3_^15^NH_4_ (60% atom ^15^N), 2.0; Na_2_HPO_4_, 1.33; K_2_SO_4_, 1.0; Cl_2_Ca, 2.0; MgSO_4_, 0.75 (mmol m^−3^) and ferric citrate, 20.0; MnSO_4_, 10.0; H_3_BO_3_, 5.0; ZnSO_4_, 1.0; CuSO_4_ (µmol m^−3^). From July 2010 onwards, pots were left unwatered receiving only natural rainfall.

Branches of every plant were collected at the start of short shoot development (25^th^ January 2010) and at the end of long shoot growth (matching blossoming, 18^st^ May 2010). The number of branches of similar size to those collected in the canopy of each plant was estimated at harvest to scale calculations up to the whole plant canopy. In all cases, branches collected accounted for a small percentage (between 1 and 8%) of the whole plant canopy, minimizing the impact of sampling on plant internal N cycling. Branches collected on each harvest were fractionated into current-year leaves (separating between green and dry), stems and inflorescences. At the end of summer drought (24^th^ September 2010), all individuals included in the experiment were harvested for isotope and chemical analyses. Roots were washed to remove sand and soil particles and individuals were separated into roots (whole system), trunks and stems ≥2 years and young branches (<2 years). A quantified subsample of the latter was further separated into green and dry current-year leaves, current-year and one-year-old stems and infrutescences. All samples were freeze-dried (Cryodos, Telstar Industrial SL, Terrasa, Spain), weighed to the closest 0.01 mg and milled in a ball mill (Restch M400) prior to analyses.

Total N content and the ^15^N signature of samples were measured by continuous flow isotope ratio mass spectrometer (Thermo Finnigan Delta Plus Advantage) interfaced to an elemental analyser (Thermo FlashEA1112, Thermo Finnigan, Bremen, Germany). Isotopic data were expressed as δ^15^N:
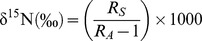
Where R_S_ and R_A_ are the molecular abundance ratios of nitrogen isotopes, ^15^N/^14^N, of the sample and international standard (Atmospheric air), respectively.

In all samples, the amount of labelled N (i.e. new N taken up throughout the experiment) was distinguished from the amount of unlabelled N (i.e. old N remobilized from storage) and calculated according to [19] with the following equations:
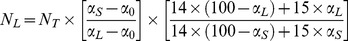


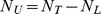
Where N_T_ is the total amount of N in the sample, and N_L_ and N_U_ are the amounts of labelled and unlabelled N, respectively, measured in unit weight (g); α_S_ and α_L_ are the ^15^N abundances (% atom ^15^N) in the sample and the nutrient solution, respectively, and α_0_ is the average natural abundance of ^15^N (% atom ^15^N) in the different organs as measured from three plants of both study species harvested in the field on the same sampling dates as in the experiment. To evaluate the relative ability of each species to uptake and remobilize N, the relative contribution (%) of labelled and unlabelled N, respectively, to the total amount of N in the canopy was calculated.

The ability of both species to recycle N from the canopy in summer was evaluated by calculating the % of recycled N in the canopy and the % of new N in total recycled N following the equations:
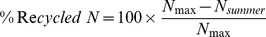


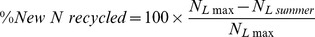
Where *N_max_* and *N_summer_* are the amount of N in the canopy of individual plants in spring (seasonal maximum) and summer, while *N_L max_* and *N_L summer_* are the amount of labelled (new) N in the canopy of plants in spring and summer, respectively.

Finally, to evaluate the ability of plants to withdraw N from senescing leaves in spring and summer we calculated the N resorption efficiency (%) (*NR_eff_*) and the N proficiency (*N_prof_*, sensu [40]) according to the following equations:
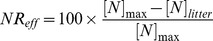


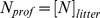
Where *N_max_* is the seasonal maximum foliar concentration of N and *N_litter_* corresponds to the N concentration of the leaf litter produced in spring or summer.

### Statistical analyses

Differences between species, fractions and sampling dates in the mass-based concentrations and pools of N were evaluated by ANOVAs with “Species”, “Fraction” and “Date” as factors. Similar models with “Species” and “Date” as factors were run for each fraction separately. To account for differences between species in the seasonal change of N concentrations in the different organs, the interaction between both factors was also included in the analyses. Finally, differences in the N concentrations among sampling dates were evaluated in each species and fraction separately by one-way ANOVAs. When significant, differences among sampling dates in the N concentrations were analyzed by the Tukey (homoscedastic residuals) or the Dunnett T3 (heteroscedastic residuals) post-hoc analysis. When the normality and homoscedasticity assumptions of ANOVA were not met, differences among sampling dates in the N concentrations of the different species were analyzed by the non-parametrical Kruskal-Wallis test (run in SPSS 15.0).

The differential ability of both species to uptake external labelled (new) N and remobilize unlabelled (old) N from storage throughout the experiment was evaluated by a repeated measures ANOVA with “Species” and “Date” as factors and the percentage of labelled and unlabelled ^15^N in the canopy of plants as response variables. Differences in the percentage of labelled and unlabelled ^15^N in the canopy of both species at given dates were evaluated by individual t-tests. The different ability of species to withdraw N from senescing leaves in spring and summer was evaluated by a repeated measures ANOVA with “Species” and “Date” as factors and *NR_eff_*, *N_prof_* and the ^15^N abundance in leaf litter (atom % ^15^N) as response variables. We also evaluated the differences between species in the % of N recycled from senescing leaves and the % of new N in the total N recycled from litter by one-way ANOVAs with “Species” as a factor.

Finally, differences between species in the % of total and new N incorporated and the total amount of N allocated to the different organs at the end of the growing season were evaluated by two-way ANOVAs with “Species” and “Fraction” as factors. Differences among fractions were evaluated separately in each species by one-way ANOVAs. Residuals of all models were checked for normality and homoscedasticity. All analyses were run in JMP 9.3 (SAS Institute, SAS9.3) unless otherwise indicated.

## Results

### Seasonal dynamics of N concentrations and pools

**Figure 1:**
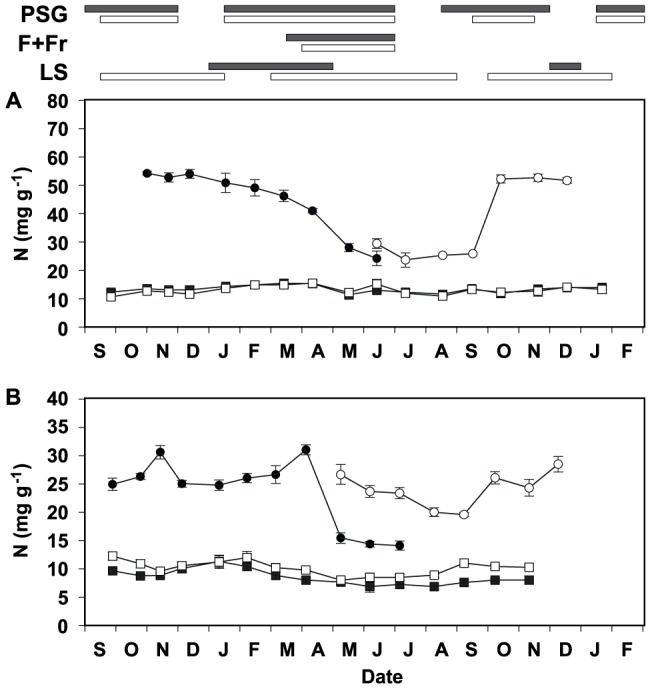
Main phenological events and seasonal dynamics of nitrogen (N) mass-based concentrations of study species. Upper bars indicate main phenological events in both species (*Lepidium subulatum*: black bars; *Linum suffruticosum*: open bars). Lower panels show N concentrations (mg g^−1^) of the short shoots (SS, round), main stems (MS, solid squares) and coarse roots (CR, open squares) of *L. subulatum* (A) and *L. suffruticosum* (B). Different cohorts of short shoots are denoted by solid (2002) and open (2003) symbols. Values are means ± SE, n = 5. PSG: primary shoot growth; F+FR: flowering and fruiting; LS: Leaf shedding.

**Figure 2:**
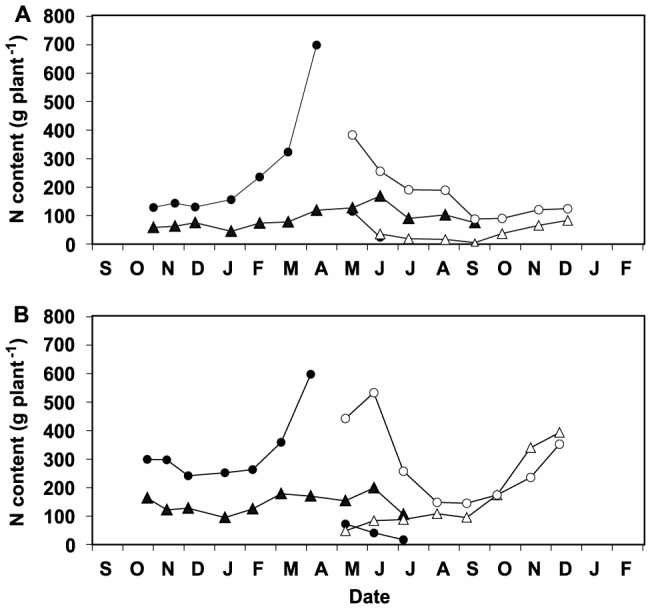
Seasonal dynamics of the nitrogen (N) content in the shoots of study species (g per plant). N content in the short shoots (whole, including both leaves and stems, SS; round) and the stems of long shoots (LS, triangles) produced in 2002 (solid symbols) and 2003 (open symbols) by *L. subulatum* (A) and *L. suffruticosum* (B) is shown. Values are the product of two means (see materials and methods for details on calculations).

**Figure 3:**
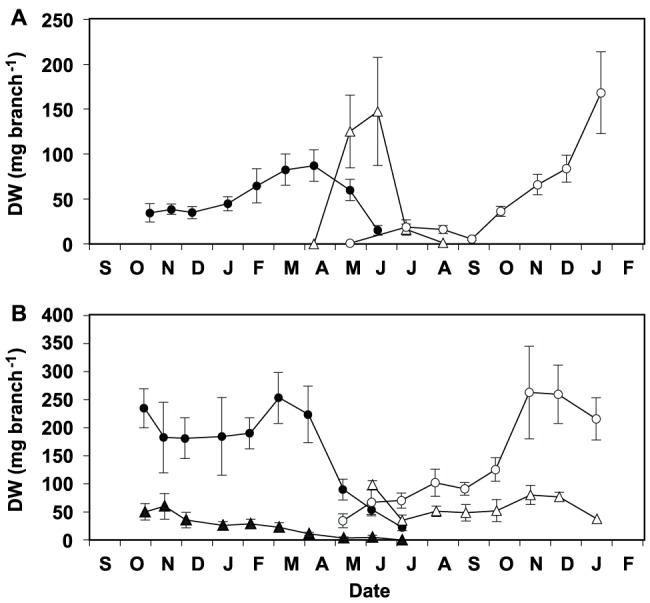
Seasonal change in the dry weight of branch components. Average dry weight of the short (SS, round) shoots and the long shoots leaves (LL, triangles) produced in 2002 (solid symbols) and 2003 (open symbols) in three-year-old branches of *L. subulatum* (A) and *L. suffruticosum* (B). Values are means ± SE, n = 15.

The foliar N concentration of *L. subulatum* almost doubled that of *L. suffruticosum* (Table 2; Fig. 1). Nevertheless, the total amount of N in foliage was similar in both species (Fig. 2), because branches of *L. subulatum* where smaller than those of *L. suffruticosum* (F_1, 1_ = 176.3, P<0.001; Fig. 3).

**Table 2:** Results of a full-factorial ANOVA testing the effects of “Species” and “Date” as on the mass-based N concentrations of the different fractions analyzed.

	Species			Date			Species x Date		
Fraction	d.f.	F	P	d.f.	F	P	d.f.	F	P
**Short shoots 02**	1	**362.0**	**<0.001**	1	**166.5**	**<0.001**	1	**25.8**	**<0.001**
**Short shoots 03**	1	**118.4**	**<0.001**	1	**78.5**	**<0.001**	1	**63.4**	**<0.001**
**Long stems 02**	1	**40.7**	**<0.001**	1	**109.1**	**<0.001**	1	2.2	0.144
**Long stems 03**	1	**29.9**	**<0.001**	1	0.004	0.948	1	2.0	0.158
**Long shoots leaves**	1	**11.1**	**0.001**	1	0.2	0.644	1	0.2	0.688
**Coarse root**	1	**69.1**	**<0.001**	1	0.9	0.340	1	3.3	0.069
**Main stems**	1	**267.7**	**<0.001**	1	**11.5**	**0.001**	1	**6.1**	**0.015**

Significant effects at α = 0.05 are highlighted in bold, n = 5.

Since leaves of short and long shoots displayed similar seasonal patterns (Fig. S1) and the latter account for a small percentage of the total leaf biomass of study species for most of the year (Fig. 3; [15]), only data of the leaves of short branches are presented. Foliar N concentrations showed significant variation throughout the year in both species, but seasonal patterns were different between them (Table 2; Fig. 1). *L. subulatum* increased foliar N concentrations in October and maintained maximal, stable N concentrations throughout autumn and winter. The N concentration of *L. subulatum* decreased when short shoots elongated into long shoots in the spring. In contrast, although foliar N concentrations of *L. suffruticosum* also peaked in autumn, values subsequently decreased throughout winter, peaking again in spring during shoot elongation (Fig. 1). Consequently and contrary to our hypothesis, the highest N concentrations were observed in short shoots during autumn in both species and, in *L. suffruticosum*, also in shoots elongating in early spring (Fig. 1). The increase in foliar N concentrations in October observed in both species was not explained by the growth phenology of their shoots, since they grew steadily between autumn and spring (Figs. 1 and 3). In contrast to *L. suffruticosum*, the autumn increase in foliar N concentrations in *L. subulatum* was not mirrored by a notable increase in foliar N pools (Fig. 2), since short shoots accounted for a relatively small proportion of the biomass during autumn and winter (Fig. 3). In both species, maximum foliar N pools (in the canopy) were attained in April-May, when the short shoots elongated into long shoots, rapidly gaining biomass (Figs.1, 2, and 3). However, the dynamics of N concentrations in the leaves differed markedly between the two species at this time of the year. In *L. subulatum*, short shoots had maintained high N concentrations throughout winter which then started to decline precisely when they elongated into long shoots (i.e. April-May, Fig. 1). In contrast, in *L. suffruticosum* there was an increase in the N concentrations of short shoots in early spring, matching the beginning of their elongation into long shoots (Fig. 1). In both species, the peak of N pools in the canopy was diluted in the following months, as the long shoots developed, flowered and dried out during late-spring and summer (Fig. 2). N concentrations of both species also declined during spring and reached the lowest values during summer (Fig. 1).

Seasonal dynamics of N mass-based concentrations in the woody organs were also different between species (Table 2; Fig. 1). *L. subulatum* showed no significant seasonal change in the N concentrations of its main stems or roots (Fig. 1; F_1,1_ = 0.375, P = 0.542, F_1,1_ = 0.385, P = 0.537 for roots and main stems, respectively). Contrastingly, N concentrations peaked in January, and in September and February in the trunks and roots, respectively, of *L. suffruticosum* (P<0.05), and minimum values were reached in June and August (P<0.05), when N concentrations of the new cohort of short shoots were hight (Fig. 1). N concentrations of young stems were also different between species (Table 2), being higher in *L. subulatum* (data not shown). However, seasonal patterns were similar across species (as denoted by the non-significant Species x Date interaction, Table 2).

### N uptake, remobilization, recycling and storage in study species

**Figure 4:**
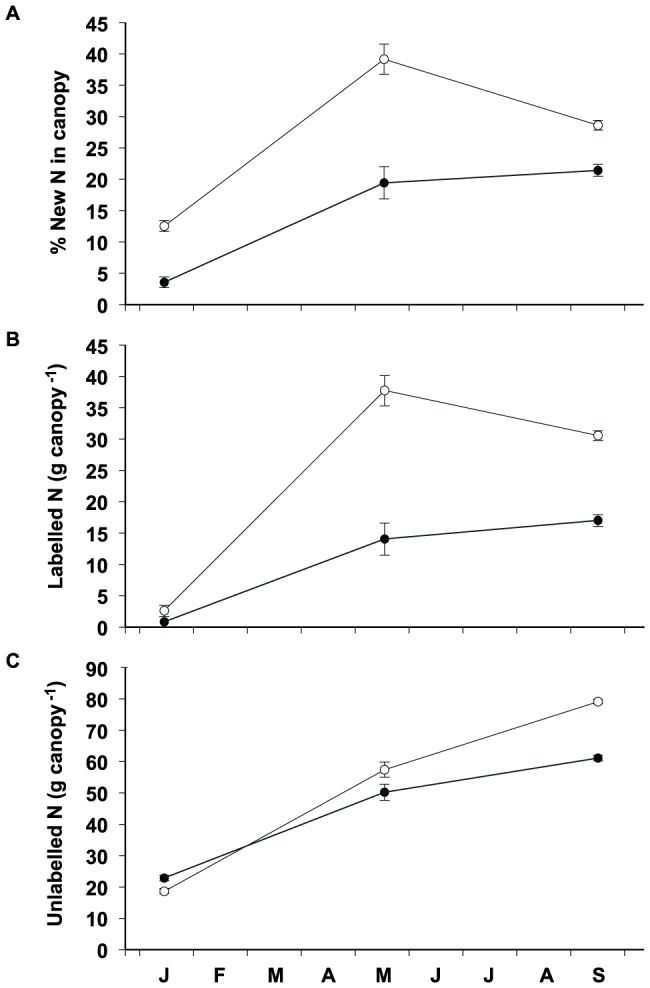
Percentage of labelled (new) N (A) and total amount of labelled (B) and unlabelled N (C) in the canopy of *L. subulatum* (open symbols) and *L. suffruticosum* (solid symbols) throughout the experiment. Data are means ± SE, n = 5.

*L. subulatum* showed a higher ability to uptake N than *L. suffruticosum*, as denoted by the higher proportion of labelled N incorporated in the canopy of cultivated *L. subulatum* plants throughout the study (Fig. 4, F_1,8_ = 97.5, P<0.001). Already in January, when the growth of short shoots was still moderate, *L. subulatum* had incorporated 67% more new N than *L. suffruticosum* (F_1,1_ = 54.2, P<0.001). Such differential new N uptake was maintained throughout the growing season and, by May (when both species were in full blossom), *L. subulatum* had incorporated 62% more new N to its canopy than *L. suffruticosum* (F_1,1_ = 30.7, P<0.001). Consequently, *L. suffruticosum* relied more on internal N remobilization than *L. subulatum* for the N supply of new shoots (Fig. 4). In both species the contribution of remobilized N decreased throughout the shoot growth period (Fig. 4) and, while unlabelled (old) N accounted for 87–96% of the total N in new shoots in winter in *L. subulatum* and *L. suffruticosum*, respectively, such proportion decreased to 60–80% in late spring. The amount of N remobilized to the canopy of both species throughout the experiment was similar (Fig. 4; F_1,8_ = 0.28, P = 0.611) and increased following shoot growth between winter and spring (F_1,8_ = 50.99, P<0.001), but remained unchanged thereafter (Fig. 4; F_1,8_ = 1.43, P = 0.266).

*L. subulatum* showed a higher ability to recycle N from the canopy during senescence than *L. suffruticosum* and the N withdrawn was mainly new N (Table 3). While *L. subulatum* recycled 60–80% of the N in the canopy between May and September and new N accounted, on average, for 60% of the total N recycled, *L. suffruticosum* showed a much variable and lower ability to recycle N from senescing organs, particularly after summer (3–58%) (Table 3). Due to the high variability among individuals of *L. suffruticosum*, differences between species in the recycling ability of new N in September were, however, not statistically significant (F_1,1_ = 0.25, P = 0.632). *L. subulatum* plants also showed higher N resorption efficiency than *L. suffruticosum* (F_1,7_ = 6.6, P = 0.037), particularly in spring, while differences between species in the N proficiency were not significant (Table 4). In general, the ability to withdraw N from senescing leaves was lower in summer than in spring for both species, as shown by the higher N proficiency values in the dry leaves produced in summer (Tables 3 and 4). The different ability of both species to withdrawn N from senescing leaves was also noticeable when comparing the isotopic composition of dry leaves produced in spring and summer (Tables 3 and 4). While the ^15^N abundance of the leaf litter of *L. subulatum* decreased from spring to summer, the opposite trend was true for the litter of *L. suffruticosum* (Tables 3 and 4).

**Table 3:** Indicators of the ability of study species to recycle N from senescing biomass between spring and summer drought (i.e. leaf litter data from May and September 2010) plus results of the ANOVA of the differences between species in the N proficiency (N prof), N resorption efficiency (NR eff) and ^15^N abundance in spring and summer leaf litter and in the % of recycled N and the % of new N in the total N recycled in the canopy between May and September 2010.

Variable	*L. subulatum*	*L. suffruticosum*	d.f.	F	P
**N prof (mg g^−1^)**					
**Spring**	0.9±0.1	0.9±0.06	1, 1	0.04	0.856
**Summer**	1.5±0.2	1.2±0.09	1, 1	2.6	0.144
**NR eff (%)**					
**Spring**	76.7±4.0	49.0±3.3	1, 1	**24.9**	**0.002**
**Summer**	58.3±12.4	36.7±2.8	1, 1	2.9	0.129
**Leaf litter ^15^N (% Atom)**
**Spring**	18.3±1.3	3.9±0.5	1, 1	**86.8**	**<0.001**
**Summer**	16.7±0.3	11.7±0.9	1, 1	**29.8**	**<0.001**
**% Recycled N**	73.8±4.4	25.1±16.9	1, 1	**7.8**	**0.027**
**% New N in recycled N**	60.6±2.7	−3.4±98.9	1, 1	0.3	0.632

Significant differences between species are highlighted in bold (n = 5).

**Table 4:** Results of repeated measures ANOVA on the effect of species and date of sampling (May and September) on the N proficiency (N_prof_), N resorption efficiency (NR_eff_) and the ^15^N abundance of leaf litter.

	Species			Date			Species x Date		
Variable	d.f.	F	P	d.f.	F	P	d.f.	F	P
**N_prof_ (mg g^−1^)**	1, 7	2.4	0.163	**1, 7**	**14.9**	**0.006**	1, 7	3.3	0.111
**NR_eff_ (%)**	**1, 7**	**6.6**	**0.037**	**1, 7**	**7.7**	**0.027**	1, 7	0.3	0.578
**Leaf litter ^15^N (% Atom)**	**1, 7**	**104.5**	**<0.001**	**1, 7**	**10.8**	**0.013**	**1, 7**	**26.3**	**0.001**

Significant effects at α = 0.05 are highlighted in bold, n = 5.

**Figure 5:**
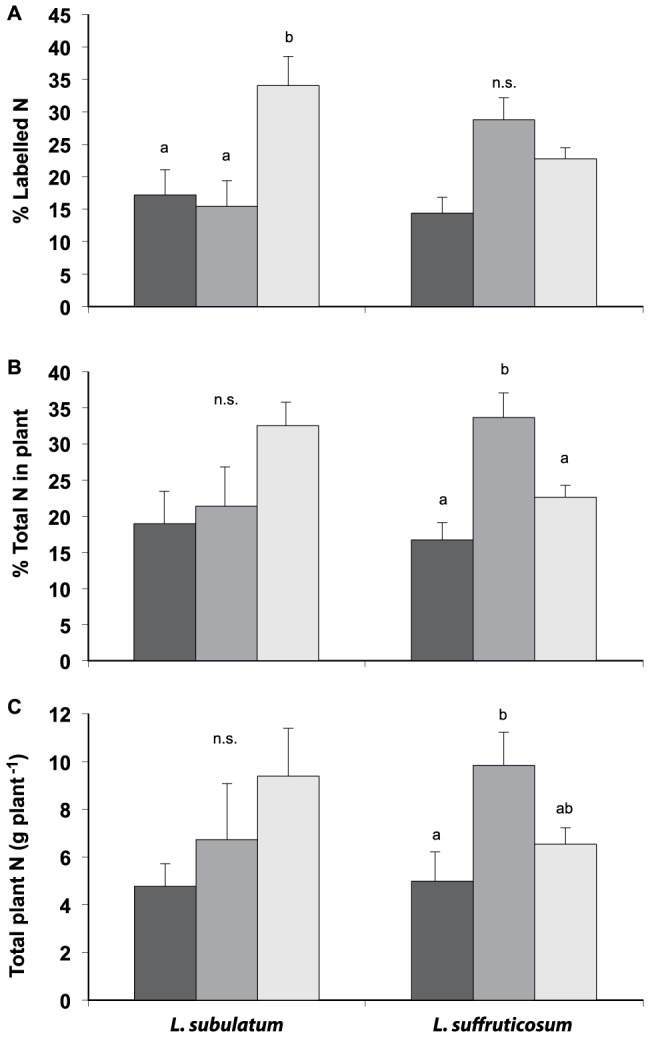
Distribution of labelled and total N at the end of the experiment (24^th^ September 2010). (A) Allocation of new (labelled) N to the main organs of both study species. (B) Distribution (% of total) of N among the main organs of study species. (C) Total amount of N allocated to the main organs of study species at the end of the experiment. Different letters indicate significant differences among fractions after one-way ANOVAs (α = 0.05). Values are means ± SE, n = 5.

Finally, we observed differences between species in the preferred organ of N storage throughout summer (Table 5, Fig. 5). Most N, including labelled N taken up throughout the current growing season, was allocated to young branches in *L. subulatum* (including leaves and young stems), while *L. suffruticosum* allocated more N to the main stem and older stems (Table 5, Fig. 5).

**Table 5:** Results of two-way ANOVAs testing the differences between species and fractions (young branches/main stems/roots) on the percentage of total and labelled N and the total amount of N allocated at the end of the growing season (September 2010).

	Species			Fraction			Species x Fraction		
Variable	d.f.	F	P	d.f.	F	P	d.f.	F	P
**Total N (g plant^−1^)**	1, 1	0.02	0.901	2, 2	3.0	0.071	2, 2	1.9	0.177
**% Total N in fraction**	1, 1	0.00	0.991	**2, 2**	**4.7**	**0.020**	**2, 2**	**4.7**	**0.019**
**% Labelled N in fraction**	1, 1	0.01	0.938	**2, 2**	**5.1**	**0.015**	**2, 2**	**4.9**	**0.016**

Significant effects at α = 0.05 are highlighted in bold, n = 5.

## Discussion

### Seasonal patterns of N concentrations were different between species and only partly explained by their leaf phenology

Our first hypothesis was only partly supported by our data since, while *L. suffruticosum* conformed to the expected seasonal pattern of maximum foliar N concentrations and pools in spring and minimum foliar N contents in summer, *L. subulatum* showed an unexpected pattern with maximum foliar N concentrations in autumn that were maintained throughout winter and further diluted by spring shoot growth (Fig. 1). The autumn maximum of foliar N concentrations in both species may be explained by a peak in external N uptake, since N mineralization reaches maximum values in semiarid gypsum ecosystems at this time of the year [41]. However, the sustained and remarkably high foliar N concentrations in *L. subulatum* throughout autumn and winter were unrelated to its shoot phenology, since shoots grew steadily from autumn to spring (Figs. 1 and 3). One plausible explanation is that *L. subulatum* stores N mainly in the old foliage, as indicated by the results of our ^15^N labelling experiment (see below). However, the strikingly high values of the foliar N concentrations of this species in autumn-winter are surprising. They more than double those of young expanding shoots (Fig. 1). Previous studies indicate *L. subulatum* has a remarkably high N, amino acid, amide and protein content [42] and a high ability to assimilate S in organic molecules [43]. Therefore, it has been suggested that this species is able to tolerate the high sulphate concentration of gypsum soils by assimilating S in N-based compounds such as proteins, peptides (glutathione), amino acids (methionine, cysteine) or other organic compounds rich in both N and S (Palacio et al., unpublished data), such as secondary metabolites (e.g. glucosinolates).

### Differences in uptake, remobilization and recycling of N

Similarly to results of previous studies on temperate winter-deciduous species [19], the summer deciduous *L. subulatum* had a higher ability to uptake external N than the evergreen *L. suffruticosum*. Although both species show a fast and pronounced ability to produce fine roots during autumn and winter [44], roots of *L. subulatum* are thinner and more intensively ramified than those of the evergreen (S. Palacio and G. Montserrat-Martí, personal observation). These traits are related to a higher ability of plants to uptake nutrients [45], which could partly explain the observed differences. The enhanced ability of the summer deciduous *L. subulatum* to uptake external N may be related to its preference for disturbed soils and its role as a pioneering species in gypsum primary succession [46], [47].

Most of the N allocated to new growth in both species came from storage. Accordingly, 60–80% of the total N of the branches of *L. subulatum* and *L. suffruticosum* at the end of the growing season came from N remobilization. These figures are similar to the 60–70% of remobilized N reported for the first pulse of growth of winter deciduous and evergreen *Vaccinium* shrubs [19]. Although both species remobilized a similar amount of stored N to supply new shoot growth, the proportion of remobilized N invested into new growth was higher in *L. suffruticosum*. A recent study on the internal N remobilization of Mediterranean species suggested that the reliance of species on remobilization from storage increases with the growth rate of plants, so that plants with high growth rate show a higher proportion of N remobilized in new shoots [27]. Interestingly, our results seem to comply with this hypothesis, since the growth rate of shoots of *L. suffruticosum* (measured in the same population used in this study) is higher than that of *L. subulatum* [36].

Although the summer deciduous species *L. subulatum* showed higher N uptake ability than the evergreen, our second hypothesis was not fully supported by our results, since the N recycling ability of the former was also higher. This was indicated by the higher % of recycled N, the higher NR_eff_ and the lower N_prof_ of the leaf litter produced in spring in *L. subulatum* as compared to *L. suffruticosum*. Consequently, the summer deciduous *L. subulatum* seems to be both more efficient in external N uptake and internal N recycling than the evergreen *L. suffruticosum*. These results are in accordance with the high N concentrations of *L. subulatum* but they contradict previous studies on Mediterranean-type ecosystems, where evergreens were more efficient in recycling N from senescent leaves than summer deciduous species [28], [29].

### The preferred site of N storage was different between species

Both the seasonal N concentrations and the total N and ^15^N allocation patterns recorded at the end of the growing season indicate that *L. subulatum* stores N preferentially in the leaves and young stems while *L. suffruticosum* does so in the trunk and main stems. These results contrast with previous studies on trees and shrubs, where winter deciduous species stored N preferentially in the wood while evergreens stored N in the old foliage [3]. One possible explanation to the differences between summer and winter deciduous species lies in the need of the former to perform opportunistic growth [29]. In continental semiarid areas, environmental conditions compatible with plant growth occur in spring and autumn and plant growth is mainly arrested during summer and also partly during winter months [15], [33], [36]. To remain competitive and complete their development, the small woody plants that grow in these environments need to be able to respond quickly and grow as soon as environmental conditions improve [36], [48], [49]. Consequently, they show a suite of traits favouring opportunistic growth such as, for example, naked buds with a short dormant period and partly preformed shoots to perform a fast shoot expansion, or short shoots with stress tolerant leaves that remain green throughout summer [34], [36], [50]. Indeed, most summer deciduous species, despite being “deciduous”, retain some of the foliage during summer (e.g. 2% of the leaves of *L. subulatum* remain green throughout summer, [15]). This contrasts with the deep rest of most temperate and boreal winter deciduous species, which remain leafless and dormant for more than 5 months. By storing N in the leaves and young stems, *L. subulatum* may be able to readily remobilize it when the first autumn rains arrive. Indeed, this species shows a relatively early autumn and spring shoot growth phenology, particularly as compared to *L. suffruticosum* [36]. The cost of accumulating N in short-lived organs such as leaves might be compensated by the relatively high (over 75%) recycling ability of *L. subulatum*. The preferred N storage in trunks and main stems of *L. suffruticosum* is harder to explain, since, although slightly delayed, this species also performs opportunistic growth and 30% of its foliage remains green throughout summer. It could be that the observed differences between study species respond simply to their different biomass allocation pattern (Fig. S2). While *L. subulatum* is a multi-stemmed plant with multiple relatively short-lived stems produced every year, *L. suffruticosum* invest ca. 50% of its biomass in the supporting (frequently single) trunk. It is hence not surprising that most of the N of this species is also accumulated in this organ.

### Conclusions

The summer deciduous *L. subulatum* shows a higher ability to uptake N than the evergreen *L. suffruticosum*, which may be related to its relatively higher foliar N concentrations and its increased ability to exploit the temporal pulses of N availability typical of semiarid ecosystems. Furthermore, *L. subulatum* is able to efficiently recycle N from senescing leaves prior to summer, accumulating it in the leaves and young stems, a strategy that may further contribute to its ability to perform fast opportunistic growth when summer drought terminates. Contrastingly, *L. suffruticosum* stores N mainly in the trunk and is less efficient in N withdrawal from senescing leaves under drought. This study provides new insights into the largely unexplored ability of semiarid shrubs to use and cycle N. The comparative analysis of species with different leaf habit indicates that multiple N cycling strategies are feasible and that they sometimes differ to those described for temperate winter deciduous and evergreen woody species, highlighting the need of further studies on semiarid and arid plants.

## Supporting Information

Figure S1 **Seasonal dynamics of the nitrogen (N) concentrations of the young fractions of study species.** Data include N mass-based concentrations of short shoots (whole, SS, round) and long shoot leaves (LL, triangles) and stems (LS, squares) of study species in 2002 (solid symbols) and 2003 (open symbols). Values are means ± SE, n = 5 except for LL that n = 3.
(EPS)

Figure S2 **Biomass allocation among the main organs of study species at the end of the experiment.** Samples were harvested on 24^th^ September 2010. Values are means ± SE, n = 5.
(EPS)
